# Supplementary material for: Repercussions faced by health professionals who have experienced the phenomenon of the second victims due to incidents related to patient safety: A scoping review protocol
Source: PLoS One. 2025 Jul 7;20(7):e0327004. doi: 10.1371/journal.pone.0327004 (PMC12233220; doi:10.1371/journal.pone.0327004)
Supplement: Attachment — Submitted filename: rebuttal letter.pdf [file pone.0327004.s003.pdf]

PLOS ONE

Rebuttal letter

To the editor and reviewers of the manuscript

Dear all,

We would like to thank you for your considerations and suggestions for improving the manuscript.

Thinking of an optimized way to answer all items, we chose to list the questions with negative answers and the considerations in the order they were presented, and sequentially we included the answers and justifications for each note.

Best regards,When submitting your revision, we need you to address these additional requirements

1. Please ensure that your manuscript meets PLOS ONE's style requirements, including those for file naming. The PLOS ONE style templates can be found at https://journals.plos.org/plosone/s/file?id=wjVg/PLOSOne_formatting_sample_main_body.pdf and https://journals.plos.org/plosone/s/file?id=ba62/PLOSOne_formatting_sample_title_authors_affiliations.pdf

The manuscript was adapted with regard to title, symbols, and authors' participation, following the indicated template.

2. Thank you for stating the following financial disclosure: [This proposal was financed in part by the Coordenação de Aperfeiçoamento de Pessoal de Nível Superior – Brazil (CAPES) – Financing Code 001.].

Please state what role the funders took in the study. If the funders had no role, please state: "The funders had no role in study design, data collection and analysis, decision to publish, or preparation of the manuscript." If this statement is not correct you must amend it as needed.

Please include this amended Role of Funder statement in your cover letter; we will change the online submission form on your behalf.

The CAPES system had no role in the study design, data collection and analysis, decision to publish, or preparation of the manuscript. This funding agency represents a government agency that funded the corresponding author's doctoral scholarship with funding code 001 (social demand).

3. Please update your submission to use the PLOS LaTeX template. The template and more information on our requirements for LaTeX submissions can be found at http://journals.plos.org/plosone/s/latex.

**Comments to the Author**

2. Is the protocol technically sound and planned in a manner that will lead to a meaningful outcome and allow testing the stated hypotheses? The manuscript should describe the methods in sufficient detail to prevent undisclosed flexibility in the experimental procedure or analysis pipeline, including sufficient outcome-neutral conditions (e.g. necessary controls, absence of floor or ceiling effects) to test the proposed hypotheses and a statistical power analysis where applicable. As there may be aspects of the methodology and analysis which can only be refined once the work is undertaken, authors should outline potential assumptions and explicitly describe what aspects of the proposed analyses, if any, are exploratory.

Reviewer #1: No

It is not clear to the authors at what point there is weakness, since the research protocol for the proposed scoping review follows the all recomendations from Joanna Brings Institute for scoping reviews.

---

3. Is the methodology feasible and described in sufficient detail to allow the work to be replicable? Descriptions of methods and materials in the protocol should be reported in sufficient detail for another researcher to reproduce all experiments and analyses. The protocol should describe the appropriate controls, sample size calculations, and replication needed to ensure that the data are robust and reproducible.

Reviewer #1: No

The methodology was rewritten, pointing out the steps proposed by JBI, the study population, the databases that will be consulted, the mnemonic used, inclusion and exclusion criteria, selection of publications, data extraction and the expected results.

---

5. Is the manuscript presented in an intelligible fashion and written in standard English?
PLOS ONE does not copyedit accepted manuscripts, so the language in submitted articles must be clear, correct, and unambiguous. Any typographical or grammatical errors should be corrected at revision, so please note any specific errors here.
Reviewer #1: No

The manuscript was reviewed by an expert in English language

Reviewer #1:
1) Ensure consistency in your writing mechanics – you use past/present/future tense in multiple sections which detracts from your main messages. There are also issues with sentence structure, typos and grammatical issues that should be addressed to improve flow and readability.

To achieve this recommendation, the manuscript was reviewed with support of an English expert speaker.

2) Abstract - Clarify the abstract. Some sections are unclear. For example, you state "Despite these advances, adverse events victims, including professionals and institutions, to progress beyond the patient." It is not clear what this sentence is conveying.

Reviewed to presente text with cohesion and clarity

3) Introduction - The introduction requires significant revisions as it was difficult to comprehend. Particular attention is needed to improve sentence/paragraph structure, organization and flow. Consider strengthening the background to build the case for why this review is needed and reduce the amount of content dedicated to explaining existing systematic/scoping reviews. The main purpose is to demonstrate that your proposed scoping review is different from what already exists in the literature.

The introduction was restructured to give cohesion, coherence and fluidity to the text.

4) Global approach - You note that your scoping review will take a global approach; however, you begin your introduction by referencing the Brazilian National Program for Patient Safety, which is very specific to one country. Consider integrating literature from a global perspective.

During the review, it was possible to identify the need for restructuring to adopt a global approach. Thus, in order to meet the review's objective, the paragraph dealing with security policy in Brazil was removed and the text was rewritten.

5) Methodology- Please provide a rationale for why the scoping review methodology (compared to other literature review methods) is the best choice for answering your questions.

Scoping review methodology is presented as proposed by JBI. Considering that the aim of the study is complex, it is understood that the approach proposed to answer the question of this scoping review is appropriate, allowing to map the repercussions arising from the second victim phenomenon in a broad, exploratory and descriptive way.

6) Review question - Your review question is: “What does the literature present about the phenomenon of second victim and the repercussions suffered by health professionals involved in incidents related to patient safety in health services”. However, in your review of the literature (on page 7) you note that there are already systematic reviews looking at the impact of adverse events on second victims. Please further clarify how your scoping review will make a different contribution to the literature.

The two reviews that are similar to this research are not contemporaneous; one was published in 2013 and the other has surgeons as its population. Considering the time that has passed and the fact that the population of this study includes all health professionals who work in health services, we can see a different result from those presented in the two reviews.

The justification for this review with these considerations is in the paragraph that follows the objective of the scoping review.

7) Language - You note that you will not limit the language – please indicate how you will review sources that are not in English or in the languages that your team is proficient in.

The following explanation has been included in the article selection section: Regarding the language of the selected articles, the reviewers are proficient in reading manuscripts in English and Spanish. For other languages, if necessary, a translation application or professional translator will be used, and the use of these mechanisms will be described in the final article.

8) Population/participants – you separate health professionals into two categories – higher education and secondary education. Please clarify this as this conceptualization may be well known across global jurisdictions. One suggestion is to state that you include both regulated and non-regulated health professionals.

Inclusion of regulated and non-regulated terms aligned with higher and mid-level professionals

9) Line 219 is unclear "the choice of the term "health professionals" instead of "health workers" is based on the definition that a health professional is a person who works in one of the professions related to health sciences, different from health professionals' health, which includes…". This sentence seems to be missing something.

In the health sciences descriptor (DeCS), "health professionals" are described as people who work in one of the professions related to health sciences, while "health workers" include, in addition to health professionals, workers who work in health service buildings, workers who work in the administrative area, pantry services, security, reception, cleaning, maintenance and who are not the focus of this study.
For clarity, a new wording was created to clarify the term used for population.

10) Line 245 subheading – please clarify what is 'types of fonts'. You may be referring to 'types of sources'

The sources are the selected studies/publications. For clarity, the term was changed and restructured within the eligibility criteria. Thus, primary and secondary studies will be included.

11) You noted that a full search was already completed on June 26th – please provide the full search as an appendix. Further, has this review already started? If the full search was conducted in June 2024, it may be worth updating the search before you commence the review to ensure the most up-to-date literature is included.

A new search was conducted on January 10, 2025

12) Sources- Please provide further information about the types of sources. For example, what type of studies (Qualitative, quantitative, mixed methods) will you include? Will you include non-empirical sources? What sources will you retrieve grey literature from? Why? what is your rationale for including such sources?

The study will use primary and secondary studies with qualitative and quantitative methodological designs, as well as mixed methods studies. Dissertations, theses and publications from societies, foundations and networks that deal with the issue of the second victim phenomenon will also be used as gray literature. The use of gray literature aims to identify works published by organizations focused on the study, updates, debate and dissemination in the area of patient safety. This section was rewritten.

13) Selection of studies – please clarify if you will be piloting the process of selecting studies to ensure members of your team are clear about the inclusion and exclusion criteria and to determine if refinements are needed.

The selection will be led by the corresponding author, this information was included in the text.

14) Data extraction – please clarify what you mean by 75% agreement (line 291-292)

This information that was disconnected from the material was removed.

15) Expected outcomes – please expand on what you anticipate to find and why. Please also clarify what you mean by “this research can contribute to the mapping of the theoretical framework on the phenomenon… (lines 326-328). Is this an existing framework?

The phenomenon of the second victim causes psychological suffering, and it is important to characterize how this suffering presents itself and what the repercussions are from this moment of professional vulnerability. In this way, this research can contribute to mapping evidence about the phenomenon.

Sincerely,

the authors

Brazil, Rio de Janeiro, February 7, 2025
