# Supplementary material for: Repercussions faced by health professionals who have experienced the phenomenon of the second victims due to incidents related to patient safety: A scoping review protocol
Source: PLoS One. 2025 Jul 7;20(7):e0327004. doi: 10.1371/journal.pone.0327004 (PMC12233220; doi:10.1371/journal.pone.0327004)
Supplement: Attachment — Submitted filename: Response to Reviewers.docx [file pone.0327004.s005.docx]

Rio de Janeiro, May 26, 2026

PLOS UM

Rebuttal Letter

Dear Editor and Reviewer of the Manuscript,

We would like to thank you for your thoughts and suggestions for improving the manuscript PONE-D-24-36642R2 “REPERCUSSIONS EXPERIENCED BY HEALTHCARE PROFESSIONALS WHO HAVE EXPERIENCED THE SECOND VICTIM PHENOMENON DUE TO PATIENT SAFETY-RELATED INCIDENTS: A SCOPING REVIEW PROTOCOL”

The adjustments to the items pointed out have been made and are listed below with the justifications for each point.

| Opinion issued | Changes/Justifications |
| --- | --- |
| 3. Is the methodology feasible and described in sufficient detail to allow the work to be replicable? Descriptions of methods and materials in the protocol should be reported in sufficient detail for another researcher to reproduce all experiments and analyses. The protocol should describe the appropriate controls, sample size calculations, and replication needed to ensure that the data are robust and reproducible.  Reviewer #1: No | We appreciate the observation. The text has been improved to meet the observation regarding the reproduction and robustness of the method. We note that the method and materials have been described in detail. That there is no sample calculation because it is a scoping review. The population, the concept and the context were described. The three steps were mentioned in the search strategy. The first stage of the search strategy was presented in the protocol, the second and third stages were described, however the result will be presented in the final article. |
| 5. Is the manuscript presented in an intelligible fashion and written in standard English?  PLOS ONE does not copyedit accepted manuscripts, so the language in submitted articles must be clear, correct, and unambiguous. Any typographical or grammatical errors should be corrected at revision, so please note any specific errors here.  Reviewer #1: No | We justify that after the last revision, points were included to meet the reviewers' needs and, unfortunately, there was no adaptation to the English language. We affirm our commitment to the written form of English and inform that the revised version has undergone corrections and adjustments so that the manuscript is clear in the proposed language. |
| 1. The abstract needs to align with changes in text – based on the revisions, gray literature will also be retrieved from Proquest Dissertations and Theses Global | Thank you for pointing out the inadequacy of the abstract. The abstract was revised and changes in the body of the manuscript were also made to the abstract. |
| 2. Introduction – “The term second victim is introduced within the patient safety framework and refers to healthcare professionals who experience psychological and professional distress following an adverse patient event” – this was previously raised and it is still not clear what ‘the patient safety framework’ entails. Is this a specific framework or are you speaking in general terms that this has been introduced in the field of patient safety? | The term "structure" is being used in general, it refers to the concepts, objectives and aspects related to the theme of patient safety. To improve the text, grammatical adjustments were made in this excerpt |
| 3. Eligibility criteria - “Additionally, within gray literature, publications cited in the reference list of the evidence synthesis will be considered if they were published by societies, foundations, or networks addressing the second victim phenomenon” – What does evidence synthesis refer to? | The synthesis of evidence according to the JBI guideline, are the registries, publications selected in the last stage of screening and eligible for analysis. In these, the reference lists will be analyzed in order to include publications that have not been captured in the search strategy and that |
| 4. Exclusion criteria – “We will exclude abstracts from scientific events, opinion articles, and publications in which the studied population comprises students from either regulated or unregulated professions.” It is not clear why abstracts from scientific events, opinion articles are separated out from publications. | In order to clarify, we reformulated the paragraph. Primary studies, opinion articles and any other publication that has students as its population will be excluded, since the objective of this research is professionals trained and inserted in the context of work. |
| 5. Exclusion criteria - Further, in your eligibility criteria, you state: “Given that a scoping review can include all types of literature 26-28, this study will consider primary and secondary studies identified in the databases used in the preliminary search.” Since you state ‘all types of literature’ it is unclear if you are excluding or including other forms of literature beyond empirical studies (e.g. peer-reviewed opinion articles, discussion papers, etc) and gray literature. | We appreciate the observation, when reviewing the text we found that it was not clear due to the writing. We revised the text to make it explicit that "primary research and reviews will be eligible, provided that they have repercussions resulting from the phenomenon of second victim in health professionals" and "abstracts of scientific events, opinion articles, and publications that did not meet the PCC and the objective of this review will be excluded. Studies on the adaptation and validation of instruments, as well as studies on the prevalence of symptoms, studies on forms of coping and forms of support that do not show the repercussions. In addition to these, publications that are not available in full will also be excluded, as well as those that exclusively addressed students from regulated or non-regulated health professions". |
| 6. Exclusion criteria - “Regarding primary sources, we will exclude those incorporated into an evidence synthesis. These publications will be identified in the third stage of the search strategy—while reviewing the reference lists of the studies included in the evidence synthesis.” This is still unclear to me. How will you know which publications are incorporated into evidence synthesis during your initial screening? | This question made us reflect and return to the manual to discuss the screening and selection process. Thus, this passage was changed based on the recommendations of the method. This stage is the third stage of the search strategy and at this point the reference lists of articles / publications included for review will be analyzed, so that any publication that has not been captured by the search strategy and that is of interest to the research is identified. Revised text: "In order to meet the third stage of the search strategy, the list of references of the selected publications will be examined after the screening carried out in the second stage and following the selection and screening path recommended for the scope reviews, the titles that meet the eligibility criteria will be manually selected. Thus, the abstracts and the full text will be read. |
| 7. Search strategy: “To ensure the inclusion of up-to-date evidence, we will perform a final search before presenting the results in the review article. This search will cover the same period and align with the last search described in the protocol. In the second step, an experienced librarian specializing in scoping review search strategies will conduct a comprehensive literature search.” It is unclear why you are conducting a final search and then a comprehensive search. Does the first ‘final search’ pertain to MEDLINE? If MEDLINE is a database that is included in your in search, it should be noted in your abstract as well. | To clarify this context, we clarify that the text has been revised. Thus, we record that a preliminary search was carried out The Virtual Health Library (VHL), Medical Literature Analysis and Retrieval System (MEDLINE), Cumulative Index to Nursing and Allied Health Literature (CINAHL) and Open Science Framework (OSF), PROSPERO and Joanna Briggs Institute (JBI) databases were found, and no manuscript or protocol record with a similar objective was found. According to the stages of the search strategy, the first stage was carried out on 01/08/2025, with a search limited to a MEDLINE database (PUBMED), and the feasibility of the proposal was verified. For the second stage, which is the survey of the databases, a librarian will be consulted to establish the search strategy. The result of the second stage will be indicated in the presentation of the research article. |
| 8. Search strategy- In the third step, we will examine the reference lists of the full-text sources selected for inclusion in the review, and publications that meet the inclusion criteria may be included”. Previously you mentioned that you would include a review of publications identified in evidence synthesis but this is not mentioned in your search strategy. | The review was carried out as mentioned in the previous items. |

**References:**

1. Peters MDJ, Godfrey C, McInerney P, Munn Z, Tricco AC, Khalil, H. Revisões de escopo (2020). Aromataris E, Lockwood C, Porritt K, Pilla B, Jordan Z, editores. Manual JBI para síntese de evidências . JBI; 2024. [https://synthesismanual.jbi.global](https://synthesismanual.jbi.global/) .
2. Sexton JB, Helmreich RL, Neilands TB, Rowan K, Vella K, Boyden J, Roberts PR, Thomas EJ. O Questionário de Atitudes de Segurança: propriedades psicométricas, dados de benchmarking e pesquisas emergentes. BMC Health Serv Res. 2006 3 de abril;6:44. <https://doi.org/10.1186/1472-6963-6-44>

We declare that the group of authors agrees with the changes made.

We remain available for any additional clarifications or adjustments that may be necessary.

Sincerely,

Elaine Cristine da Conceição Vianna Gonçalves da Costa

*Corresponding author*
